# Abortion Access Persists, but So Do the Threats

**DOI:** 10.1002/hast.70043

**Published:** 2026-02-04

**Authors:** Katie Watson

**Keywords:** shield laws, state abortion laws, right to travel, medication abortion, telemedicine, reproductive ethics

## Abstract

“Shield laws” declare that, for purposes of reproductive health care, the law of the jurisdiction in which the clinician practices governs when state laws conflict. In 2024, approximately 100,000 pregnant people living in states that criminalize abortion provision received pills for a medication abortion from a clinician living in one of the eight states with these laws. One of these clinicians is New York's Margaret Carpenter, who was criminally charged in Louisiana and fined and enjoined in Texas. Carpenter's case testing shield laws, which is likely to go to the U.S. Supreme Court, should be framed as a “right to travel case” because telemedicine should be understood as a modern version of travel. If the Supreme Court ultimately accepts Louisiana and Texas's likely argument that it's a narrow “state regulation of medicine” case, the Court will be limiting the constitutional right to travel to people who have the money and time to physically travel for medical care, and withholding it from people who need the same care but who can afford to access it only through virtual travel.

## Perspective

In 2022, the reversal of *Roe v. Wade* was terrible news for those who value reproductive autonomy. Three years later, the surprising news is that, so far, the legislation in nineteen states to criminalize abortion provision before viability has failed to reduce the total number of abortions in the United States.

In 2024, approximately 25 percent of U.S. abortion patients lived in states where abortion provision is banned. According to the Society for Family Planning's *#WeCount* report, approximately 140,000 of these patients traveled to an access state for care, while roughly 100,000 stayed in their home state for a telemedicine appointment with a clinician in an access state and then received the pills they were prescribed for a medication abortion by mail.

The success of this medical rescue effort is why Texas and Louisiana went after New York physician Maggie Carpenter this fall in a test of “shield laws.” Carpenter's case is likely to become the Supreme Court's next blockbuster ruling on abortion, and if the Court invalidates these laws, the criminalization of abortion provision will inflict a new level of devastation. A less obvious reason bioethicists should be watching this case is that it's about the regulation of new medical technologies, as well as economic justice in medical access.

Shield laws were passed after *Roe* was reversed by eight states that protect abortion access. These laws declare that, for purposes of reproductive health care, when state laws conflict, the law of the jurisdiction in which the clinician practices governs. In addition, they declare that the clinician's state will not follow the traditional practice of serving out‐of‐state subpoenas and judgments on its residents if the basis of the other state's charge is providing reproductive health care that is legal in the clinician's state. A Louisiana grand jury indicted Carpenter on a criminal charge that carries a minimum of one and up to five years in jail, and a Texas court levied a $113,000 fine and an order to stop mailing medication abortion after Carpenter sent it to patients in Louisiana and Texas. However, because medication abortion is legal under New York's Reproductive Health Act, New York has refused to extradite or serve the financial judgment on Carpenter.

Louisiana and Texas will argue that this is a narrow “state regulation of medicine” case, but it's much bigger than that. The constitutional basis for our right to travel between states is independent of how we travel. That has evolved from horses to cars to airplanes, and telemedicine should be understood as a modern version of travel. In the physical version of medical travel, a patient initiates contact, receives the clinician's consent to make an appointment, and gets themselves to the clinician's state for treatment. In the virtual version of medical travel, a patient does the same thing yet arrives over the internet. Prescribing for medication abortion requires only a verbal interview, not a physical exam. If the Court rules that the patient's jurisdiction is entitled to govern telemedicine in all circumstances, it will be limiting the constitutional right to travel to people with the economic ability and life circumstances that permit physical travel for medical care and withholding it from people who need the same care but who can afford to access it only through virtual travel.

The grit of hundreds of thousands of women in ban states fighting to obtain the life‐altering health care they need and the resolve of clinicians taking risks to provide them the access everyone deserves should inspire admiration, not a belief that we can live with abortion bans. No one should have to reach outside their home state for medical care, and new research shows that many travelers experience catastrophic health expenditures, psychological harm, and loss of confidentiality due to the need to ask people for financial and logistical help. In reporting that has generated disappointingly little public outcry, Pro Publica has documented six deaths attributable to the bans. And bans also insult the personhood of the three out of four women who reach menopause without wanting or needing an abortion but know their state didn’t want that decision to be theirs to make.

However, the fifty‐third anniversary of *Roe* in January 2026 remains a good time to celebrate that those who seek to eliminate women's self‐determination haven’t won yet, and to understand that the resolution of Carpenter's case will help to determine whether that remains true.

